# Forewarned is forearmed: Queensland fruit flies detect olfactory cues from predators and respond with predator-specific behaviour

**DOI:** 10.1038/s41598-020-64138-6

**Published:** 2020-04-29

**Authors:** Vivek Kempraj, Soo Jean Park, Phillip W. Taylor

**Affiliations:** Applied BioSciences, Macquarie University, Sydney, NSW Australia

**Keywords:** Ecology, Behavioural ecology, Evolutionary ecology

## Abstract

Animals can gain significant advantages from abilities to detect cues from predators, assess risks, and respond adaptively to reduce the likelihood of injurious interactions. In contrast, predator cue-induced changes in behaviour may interfere with fitness-associated activities such as exploration, foraging and reproduction. Despite the ecological importance of predator-prey interactions in insects, remarkably little is known about the abilities of insects to detect and respond to olfactory cues from predators, or the potential costs of such responses. We here demonstrate that a tephritid fruit fly, the Queensland fruit fly *Bactrocera tryoni*, is able to detect and respond differentially to volatile olfactory cues from four potential predators (three spiders and an ant) that vary in prevalence and diurnal activity. Male and female flies increased or decreased motility (velocity, active time, distance moved), or exhibited no change in motility, depending on which predator volatiles they encountered. Further, flies significantly reduced foraging, oviposition and mating propensity in the presence of volatiles from any of the predators. This study is the first report of predator-specific responses to olfactory cues in a tephritid fruit fly, and highlights that such anti-predator responses can impose costs on general activity and reproductive behaviour.

## Introduction

Direct physical encounters between predators and prey are the most apparent elements of predator-prey interactions. However, there are also myriad less visible ways in which predators and prey affect each other, modifying their behaviour in response to predatory opportunities and predation risks, respectively. Animals commonly emit or deposit characteristic chemical signatures that can be exploited as valuable sources of information by their predators. Diverse predators use olfactory cues to locate and identify prey. For example, the German wasp, *Vespula germanica*, uses the pheromone produced by male Mediterranean fruit flies as a cue to locate its prey^1,2^. Similarly, a Zodariid spider *Habronestes bradleyi* is attracted to alarm pheromones of its ant prey, *Iridomyrmex purpureus*^3^. On the other hand, prey species may detect olfactory cues from predators, and use this information to modify their behaviour to reduce risks of attack^4–6^. Such ‘non-consumptive effects’ of predators on prey behaviour are crucial for understanding ecological structure of predator-prey interactions^7^. Although numerous studies exist for olfaction-mediated indirect effects of predators on prey in vertebrates and aquatic systems^8–12^, much less is known about terrestrial insect systems^13,14^.

Responses of prey species to cues from their predators have been investigated in several insect systems. Aphid colonization is decreased in the presence of contact chemical cues from *Coccinella septempunctata* ladybirds^15^. Similarly, Colorado potato beetle larvae, *Leptinotarsa decemlineata*, reduce feeding in the presence of predator cues^14^. Striped cucumber beetles, *Acalymma vittatum*, reduce feeding and movement in the presence of tactile and visual cues from wolf spiders^16^. Most studies of non-consumptive effects in insect systems have focused on sensory modalities other than olfaction and have reported effects of cues from a single predator. However, insects may be attacked by diverse predators, each of which may present different risks and may have a characteristic chemical signature^17^. There are numerous contexts within which responses to olfactory cues from predators, and trade-offs, might be anticipated. Motility is important for most insects^18^, and inhibition of motility can have deleterious impacts on fitness. Because of differences amongst predators in sensory abilities and behaviour, prey species might move more quickly, or slower, or freeze, depending on the type of predator they encounter^4^.

Foraging is vital for survival and reproduction, but animals may be confronted with the challenge of acquiring food under increased risk of predation. Such conflicts in fitness currencies result in a trade-off to which prey may respond to predator cues by reducing foraging activities^19,20^. Mating is commonly associated with increased predation risk^21^. Sexual signals not only broadcast an individual’s location or quality to potential mates, they can also alert predators to the location of potential prey^1,22,23^. Oviposition in insects is often a complex process that involves active site-selection and egg laying that may make females more vulnerable to detection or attack. Insect eggs are easy targets for predators, and so female insects may appraise the suitability of an oviposition site in order to reduce predation risks for their offspring^24^. Although there are many studies investigating oviposition behaviour in the presence of predators or other dangers^25,26^, the effects of olfactory cues from predators on oviposition by insects are little known. Given differences among predators in the risks posed to prey species, and in prey responses that might mitigate risk, understanding the effects of olfactory cues from diverse predators is essential to progress beyond understanding prey response in single predator-prey systems.

In this study, we assess the ability of predator-naïve Queensland fruit flies, *Bactrocera tryoni* (Froggatt) (Tephritidae), to detect and respond to olfactory cues from four taxonomically and ecologically diverse cursorial predators and highlight the impact of their responses on fitness-related behaviours, including motility, feeding, and oviposition. *Bactrocera tryoni* is highly polyphagous, developing in more than 100 different fruits, and is widespread in Eastern Australia, having dispersed far beyond its pre-European range over the past century and also having established in some Pacific islands^27,28^. Tephritid fruit flies are ecologically and economically important in most tropical and warm temperate regions of the globe, and yet very little is known about their relationships with predators or their abilities to detect and respond to predation risk. The present study is an important step in advancing understanding of predator-prey interactions in tephritid flies, as well as in insects more broadly.

## Results

### Flies alter motility when exposed to olfactory cues from predators

In the presence of olfactory cues from predators, non-predators, and filtered air controls female and male flies exhibited significant variation in (i) mean velocity (Females χ^2^ = 149.3, *df* = 29, *P* < 0.0001; Males χ^2^ = 120.5, *df* = 29, *P* < 0.0001; Fig. 2A,B), (ii) active time (Females χ^2^ = 80.2, *df* = 29, *P* < 0.0001; Males χ^2^ = 81.4, *df* = 29, *P* < 0.0001; Fig. 2C,D), and (iii) distance moved (Females χ^2^ = 153.6, *df* = 29, *P* < 0.0001; Males χ^2^ = 140.4, *df* = 29, *P* < 0.0001; Fig. 2E,F). Compared to flies exposed only to filtered air, female and male flies significantly increased velocity, active time, and distance moved in the presence of olfactory cues from *H. minitabunda* and *O. smaragdina*, but showed significantly reduced velocity, active time and distance moved when exposed to olfactory cues from *C. robusta* (Fig. 2). In contrast, velocity, active time and distance moved of female and male flies in the presence of olfactory cues from *O. quadratarius* and a non-predator, *P. affinis*, were not significantly different from control (Fig. 2).

**Figure 1:**
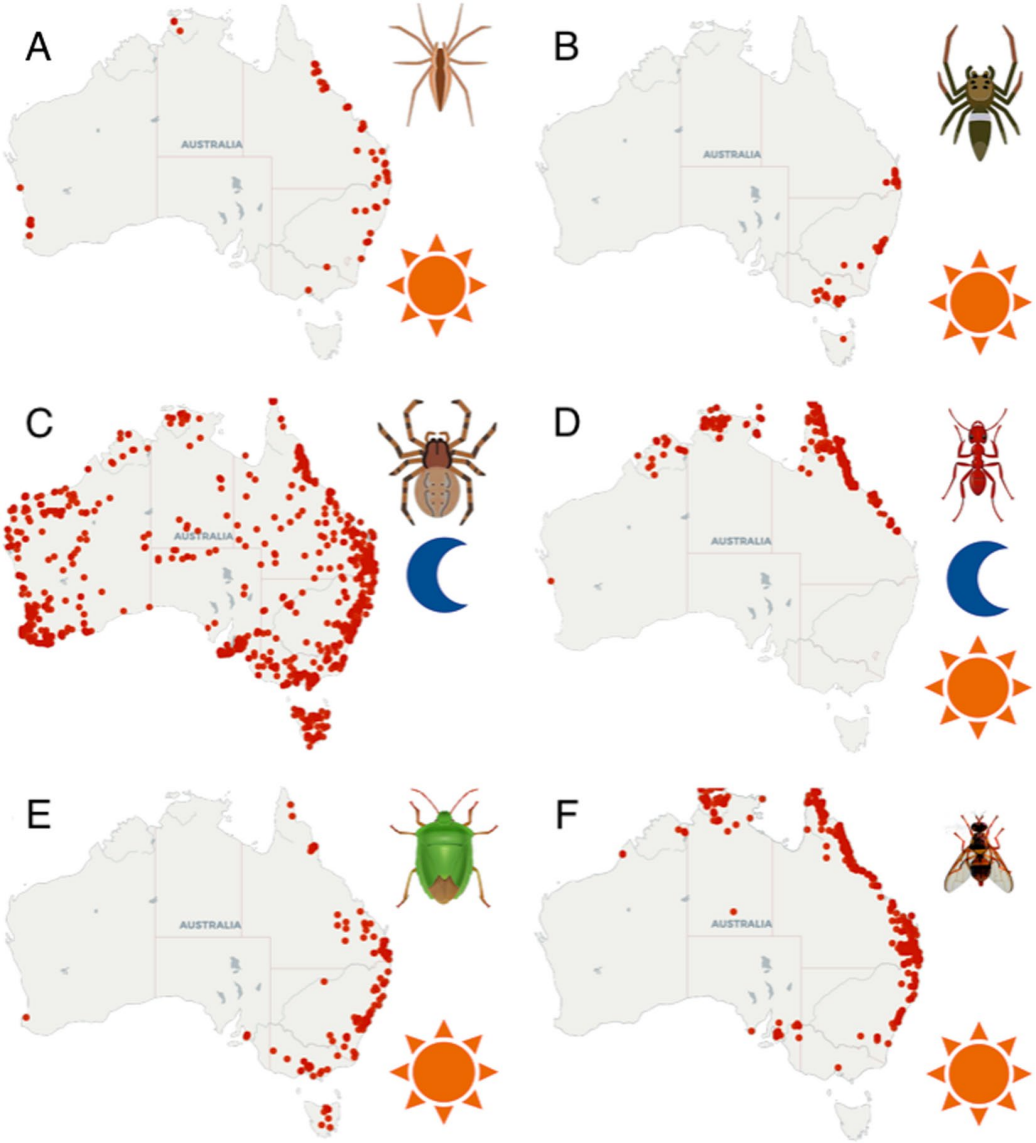
Distribution and diurnal activity of predators, prey and non-predator. (**A**) *Helpis minitabunda* a tropical/sub-tropical spider and is mostly found in the north eastern region of Australia. It is a diurnal jumping spider (depicted with yellow sun). (**B**) *Opisthoncus quadratarius* is a temperate spider found towards the south eastern region of Australia and is a diurnal jumping spider. (**C**) *Clubiona robusta* is a cosmopolitan clubionid spider with a wide range. It is present throughout Australia and is nocturnal (depicted with a blue moon). (**D**) *Oecophylla smaragdina* is commonly known as the green tree ant and is mostly present in the northern regions of tropical Australia. Workers are active in the day as well as night (depicted with both yellow sun and blue moon). (**E**) *Plautia affinis*, the green stink bug is common in gardens and orchards of eastern Australia. It is an herbivorous sap-feeding non-predator and is diurnal. (**F**) *Bactrocera tryoni* is a fruit fly native to eastern Australia and is also known as the Queensland fruit fly. Previously its range was limited to north eastern Australia, but its range has expanded since European colonisation. Distribution data are from atlas of life, Australia (atlasoflife.org.au). Illustrations are by V.K.

**Figure 2:**
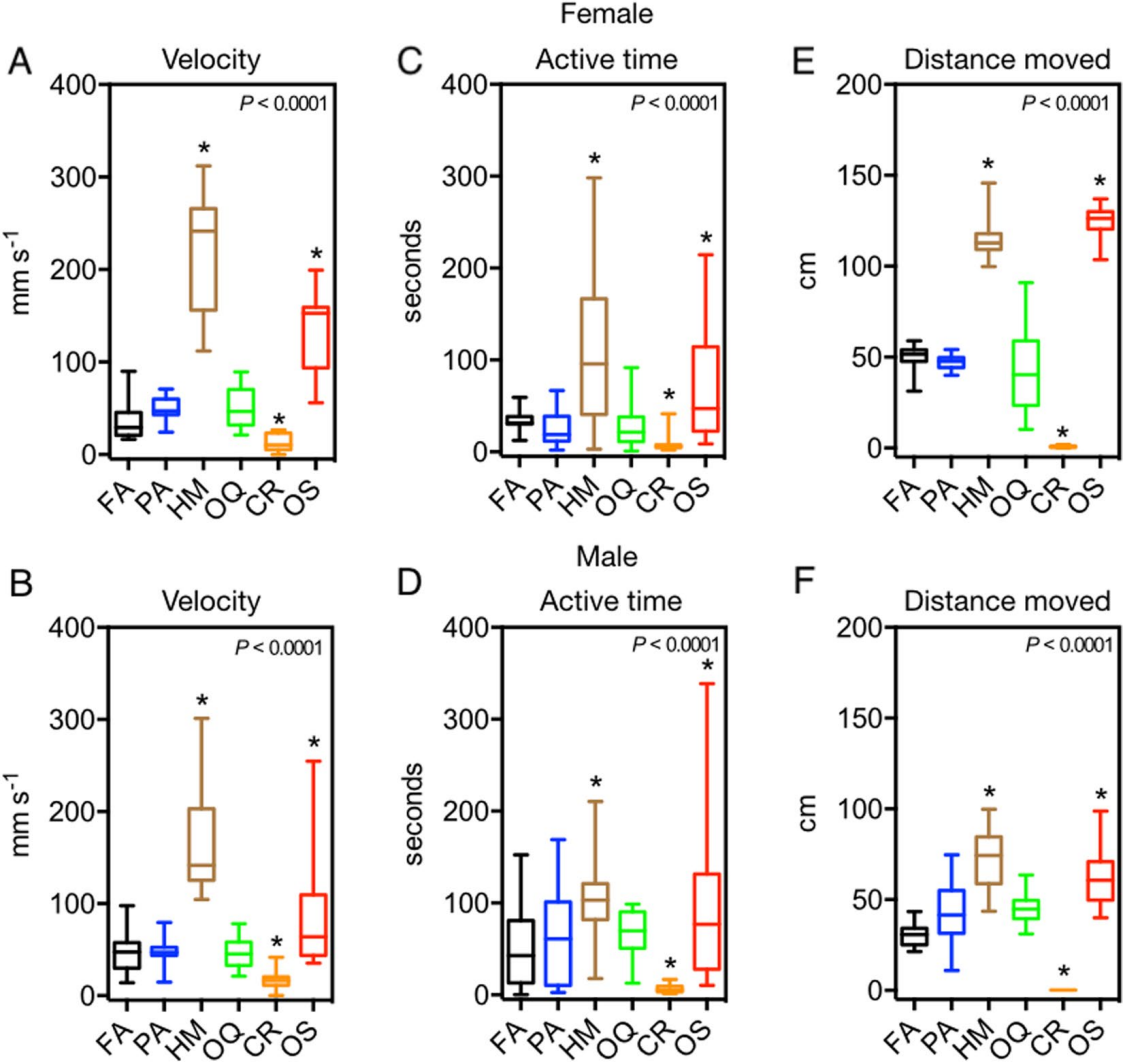
Motility of *B. tryoni* exposed to predator olfactory cues. Flies either increased or decreased motility, measured by flies (**A**,**B**) velocity, (**C**,**D**) active time and (**D**,**E**) distance moved, depending on the predator that cues were from. Differences across the set of treatments was analysed by Kruskal-Wallis test (*P* < 0.0001, see text) followed by Dunn’s multiple comparison test against filtered air control. Asterisk denotes significant difference from control and whiskers denote the max and min. FA = Filtered Air; PA = *Plautia affinis*; HM = *Helpis minitabunda*; OQ = *Opisthoncus quadratarius*; CR = *Clubiona robusta*; OS = *Oecophylla smaragdina*.

### Flies decrease foraging when exposed to olfactory cues from predators

**Figure 3:**
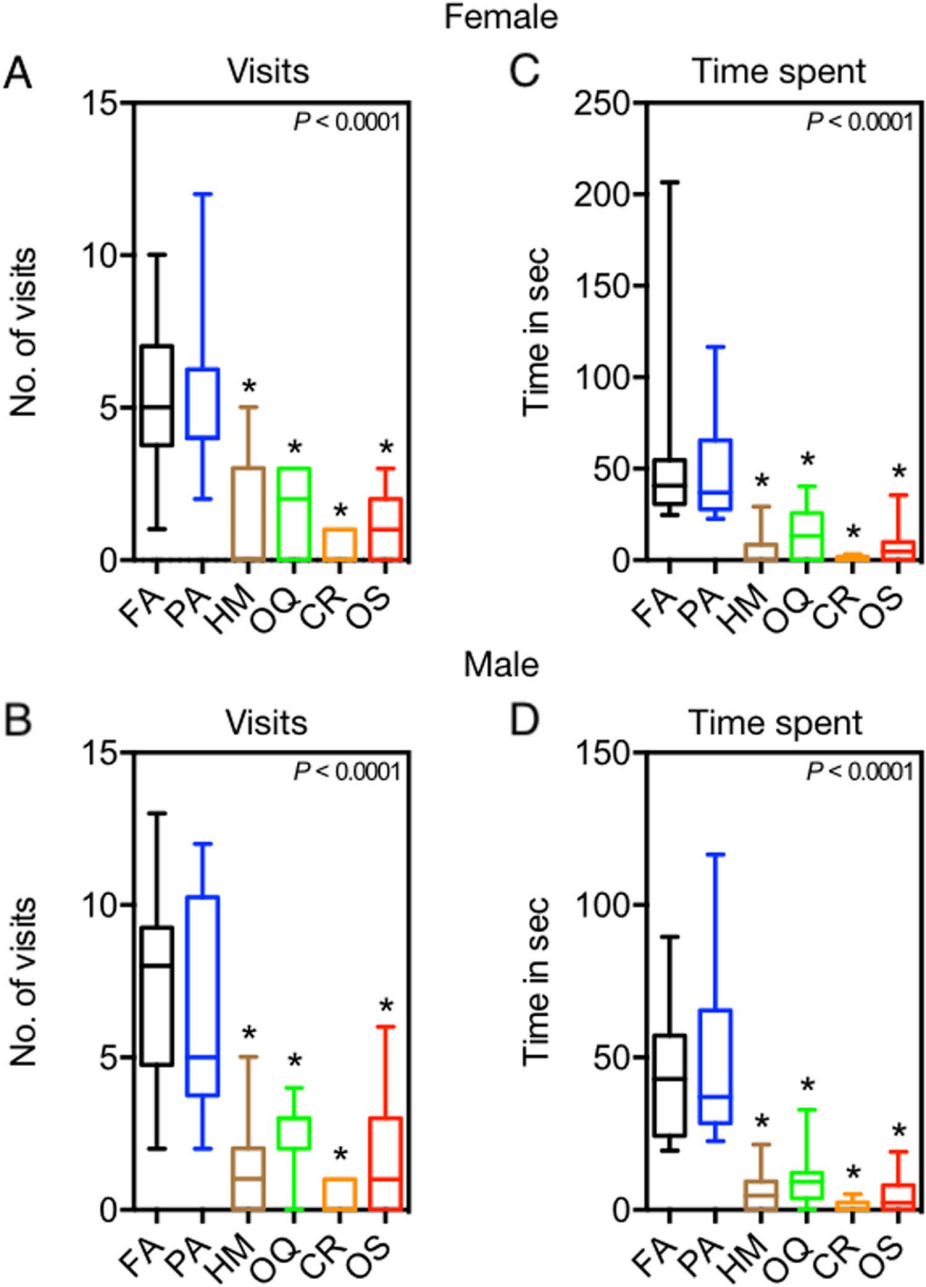
Foraging by *B. tryoni* exposed to predator olfactory cues. Flies decreased foraging, measured by (**A**,**B**) visits and (**C**,**D**) time spent. Differences across the set of treatments was analysed by Kruskal-Wallis test (*P* < 0.0001, see text) followed by Dunn’s multiple comparison test against filtered air control. Asterisk denotes significant difference from control and whiskers denote the max and min. FA = Filtered Air; PA = *Plautia affinis*; HM = *Helpis minitabunda*; OQ = *Opisthoncus quadratarius*; CR = *Clubiona robusta*; OS = *Oecophylla smaragdina*.

Number of visits to a food zone by female and male flies varied significantly in the presence of olfactory cues from predators, non-predators, and filtered air controls (Females χ^2^ = 111.3, *df* = 29, *P* < 0.0001; Males χ^2^ = 113.5, *df* = 29, *P* < 0.0001; Fig. 3A,B). Both female and male flies made fewer visits to the food zone when exposed to olfactory cues from the predators *H. minitabunda*, *O. quadratarius*, *C. robusta*, and *O. smaragdina* compared to flies exposed only to filtered air. However, number of visits to food zones in the presence of olfactory cues of non-predator *P. affinis* was not significantly different from control. Time spent in the food zone by female and male flies varied significantly in the presence of olfactory cues from predators, non-predators and filtered air controls (Females; χ^2^ = 124.1, *df* = 29, *P* < 0.0001; Males χ^2^ = 131.7, *df* = 29, *P* < 0.0001; Fig. 3C,D). Reflecting results for number of visits to the food zone, both female and male flies spent less time in the food zone when exposed to olfactory cues from *H. minitabunda*, *O. quadratarius*, *C. robusta*, and *O. smaragdina* compared to flies exposed only to filtered air. However, amount of time in the food zone in the presence of olfactory cues of non-predator *P. affinis* was not significantly different from control.

### Flies reduce oviposition in the presence of olfactory cues from predators

**Figure 4:**
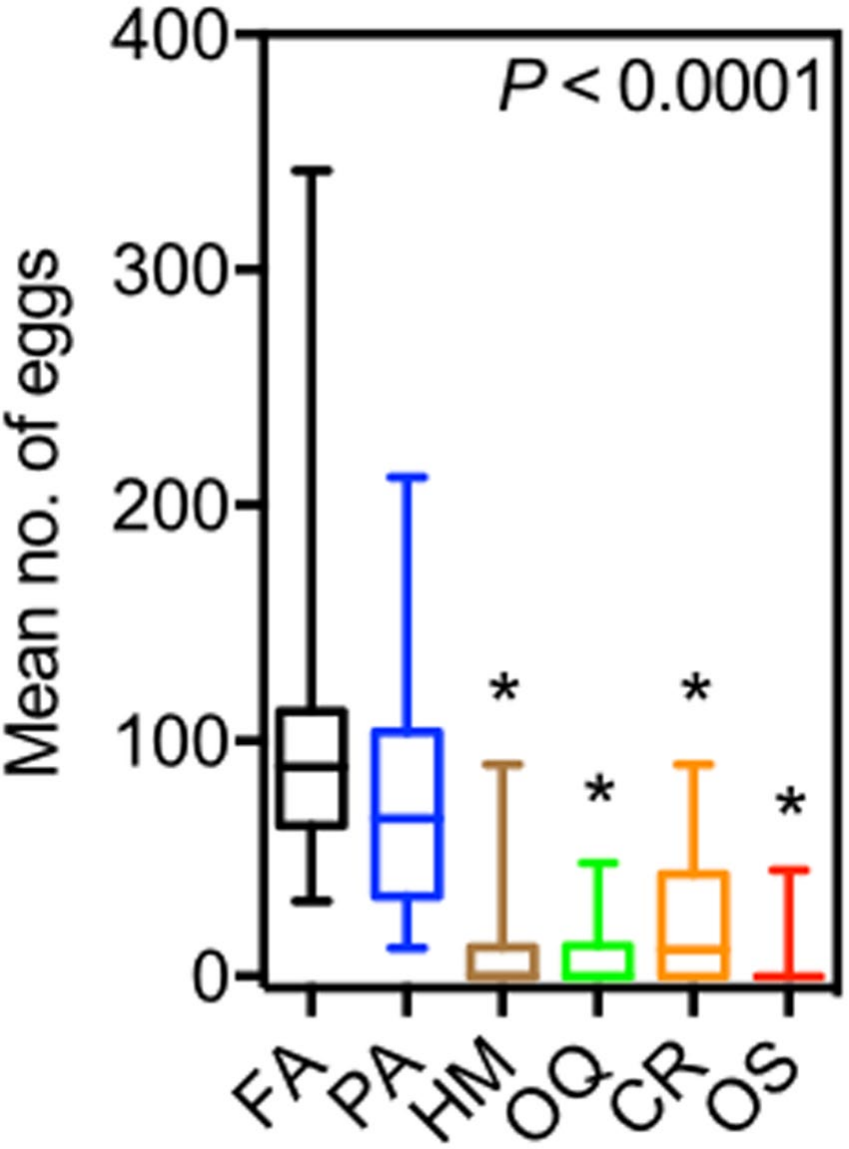
Oviposition by *B. tryoni* exposed to predator olfactory cues. Flies decreased overall oviposition in the presence of predator cues. Differences across the set of treatments was analysed by Kruskal-Wallis test (*P* < 0.0001, see text) followed by Dunn’s multiple comparison test against filtered air control. Asterisk denotes significant difference from control and whiskers denote the max and min. FA = Filtered Air; PA = *Plautia affinis*; HM = *Helpis minitabunda*; OQ = *Opisthoncus quadratarius*; CR = *Clubiona robusta*; OS = *Oecophylla smaragdina*.

Differences were found in the number of eggs laid by *B. tryoni* in the presence of olfactory cues of predators, a non-predator, and filtered air (χ^2^ = 116.1, df = 29, *P* < 0.0001). Female *B. tryoni* laid significantly fewer eggs in the presence of olfactory cues from all tested predators compared to control (Fig. 4). Female *B. tryoni* laid 106.6 ± 12.30 eggs (mean ± s.e.m) in the presence of filtered air (control), but laid significantly fewer eggs in the presence of olfactory cues from *H. minitabunda* (9.1 ± 3.6 eggs), *O. quadratarius* (23.60 ± 5.04 eggs), *C. robusta* (7.96 ± 2.31 eggs), *O. smaragdina* (2.76 ± 1.61 eggs); however, number of eggs laid by flies in the presence of cues from non-predator *P. affinis* (78.90 ± 10.24 eggs) was not significantly different from control.

### Flies do not mate when exposed to predator cues

When exposed only to filtered air all 30 tested pairs mated, attempting copulation 4.23 ± 0.23 times before success and copulating for 63.72 ± 10.23 min (mean ± s.e.m). In the presence of non-predator olfactory cues, 20 of the 30 pairs mated (67%, a significant reduction compared with filtered air; Fishers exact test *P* < 0.001), attempting copulation 6.34 ± 1.34 times before success (not significantly different from filtered air; Welch ANOVA *F*_1,31.99_ = 3.285, *P* = 0.079) and copulating for 43.45 ± 7.34 min (not significantly different from filtered air; Welch ANOVA *F*_1,47.29_ = 2.061, *P* = 0.158). In contrast, no flies were observed to attempt copulation in the presence of olfactory cues from any of the predators (a significant reduction compared with both filtered air and non-predator olfactory cues; for both Fishers exact test *P* < 0.001 in all comparisons) and all flies spent the vast majority of time standing still. Although not quantified, male calling behaviour (rapid wing fanning)^29^ was commonly observed in the presence of filtered air or non-predator olfactory cues, but was never observed in the presence of predator olfactory cues.

## Discussion

This study demonstrates very substantial effects of predator olfactory cues on motility, foraging, oviposition and mating activity of *B. tryoni*, as well as comparatively modest effects of non-predator cues on oviposition and mating. Olfactory cues from predators increased or decreased fly motility, depending on predator species. Specifically, *B. tryoni* did not modify motility level in the presence of olfactory cues from the spider *O. quadratarius* (or from the non-predator, *P. affinis*) but showed substantial changes in motility in response to cues from other spiders, *H. minitabunda* (increase), *C. robusta* (decrease), and an ant, *O. smaragdina* (increase). The lack of motility response to olfactory cues from *O. quadratarius* appears not to reflect a lack of detection, as cues from this species significantly inhibited foraging, oviposition and mating. The strong motility response to cues from *H. minitabunda*, *C. robusta* and *O. smaragdina* may reflect that these species are much more prevalent as leaf-dwelling predators in the ancestral range and habitat of *B. tryoni*, whereas *O. quadratarius* is less common in the ancestral range and is usually found on exposed tree trunks and branches of trees rather than on leaves (Fig. 1). Comparable ability to discriminate and respond differentially to cues from diverse predators has been reported in wood crickets and wolf spiders^4,30^, but not in tephritid fruit flies. Some vertebrate and insect species are known to respond to various predators based on particular predator traits, such as size, and commonness^4,31^. However, the present study appears to be the first to show that an insect, *B. tryoni*, can respond to olfactory cues of various predators with either an increase or decrease in motility. Previous studies have consistently found decreases in prey motility after detection of predator cues. This may be because these studies focused on nocturnal predators^30,32–35^. Nocturnal predators tend to rely less on vision, and more on substrate vibrations generated by moving prey to discern prey presence and location and this may have promoted a response of reduced motility when exposed to cues from nocturnal predators^4^. When in the presence of a nocturnal predator, decreased motility may increase the chances of remaining undetected^36^. However, decreased motility may be a less appropriate response in daytime by increasing vulnerability to visually orienting diurnal predators^37,38^. During the day, when the flies are able to rapidly decamp by visually oriented locomotion or flight, increased motility may be a more effective strategy to avoid non-flying predators^31,39,40^.

*Bactrocera tryoni* exhibited reduced number of visits and time spent at a food source in the presence of olfactory cues from predators. Reduced foraging activity has an obvious fitness cost of reduced acquisition of nutritional resources^41^. The absence of response to olfactory cues from the tested non-predator indicates that the response to predator cues does not reflect simply a general change in foraging behaviour in the presence of any olfactory cues. Instead, the reductions in foraging behaviour appear to reflect the danger indicated by specific olfactory cues from predators.

Animals may accrue substantial fitness advantages when they choose to oviposit in sites that increase the survival and growth of their offspring^24,26^. There is abundant evidence that many aquatic insects make oviposition choices based on cues that enable them to estimate predation risk, thus decreasing mortality of their offspring^42^. However, few studies in terrestrial insects are available. As predicted, female flies decreased oviposition in the presence of predator cues. Effects of both ant and spider volatiles likely reflect direct risks to ovipositing female flies. Oviposition requires a female to spend time at an oviposition site, likely increasing the chances of detection by a predator. Also, female flies may be less able to detect or escape from predators while ovipositing. Responses to olfactory cues from the ant *O. smaragdina* may also reflect that ants forage on fruit fly eggs and larvae in addition to adults^43^.

Olfactory cues from predators completely eliminated mating activity of *B. tryoni*. In some wolf spiders, males are known to wait longer to initiate mating in the presence of predator cues, thereby decreasing predation risk^44^. While this change in mating behaviour reduces predation risk, males employing this strategy often suffer reduced mating rates^23^. Only a handful of previous studies have reported differential responses of prey species to odors from different predators^14,45^ with no previous studies in tephritid flies. Chemical cues from predators that drive non-consumptive effects are poorly understood in insects (For an exception, see^25^). Some studies have reported that prey species respond to predator cues associated with consumed conspecifics, rather than endogenous predator cues^10,19,46,47^. In our experiments, predators were not fed *B. tryoni*, and hence the olfactory cues appear to be from the predators themselves. The flies used in our experiments were predator-naïve, and so the observed responses must be innate rather than learned.

This is the first detailed study of predator-specific responses to olfactory cues in a tephritid fly and is one of few such studies in terrestrial insects more broadly. Our findings indicate olfaction-mediated behaviour as a key for understanding predator-prey relations in tephritid flies, as well as for how tephritid fruit flies manage conflicts and trade-offs between fitness currencies. We found distinct responses to olfactory cues from each of the predators tested with *B. tryoni* and anticipate that distinct responses would also be evident for many other untested predators. We also anticipate that other tephritid flies will have comparable abilities to detect and respond to olfactory cues from their similarly diverse predators. The present study indicates that substantial advances in understanding the behavioural and chemical ecology of tephritid flies can be achieved by a new focus on the role of olfaction in predator-prey relations.

## Methods

### Study system

Queensland fruit fly, *Bactrocera tryoni* (Diptera: Tephritidae) (Fig. 1F), were obtained from a colony originating from central coastal New South Wales (29 generations from wild, at ~5,000 flies per generation) and maintained in a controlled environment laboratory at Macquarie University (25 ± 0.5 °C, 65 ± 5% RH, photoperiod of 11.5:0.5:11.5:0.5 light: dusk: dark: dawn). Adult flies were fed yeast hydrolysate, sugar and water *ad libitum*. Four native cursorial predators - three spiders and an ant – and one native non-predator were selected as sources of olfactory cues. The distribution and diurnal activity of predators, prey and non-predator are presented in Fig. 1. Three spiders, *Helpis minitabunda* (Salticidae, Fig. 1A), *Opisthoncus quadratarius* (Salticidae, Fig. 1B), *Clubiona robusta* (Clubionidae, Fig. 1C), and an ant *Oecophylla smaragdina* (Fig. 1D), were used as sources of predator olfactory cues. Female spiders were collected from December 2017 to March 2018 from orange orchards of NSW Department of Primary Industries, Somersby, NSW, Australia, and workers of *O. smaragdina* were obtained from August to October 2018 from trees around the Department of Agriculture and Fisheries, Mareeba, QLD, Australia. Spiders were maintained in the controlled environment laboratory (25 ± 0.5 °C, 65 ± 5% RH, photoperiod of 11.5:0.5:11.5:0.5 light: dusk: dark: dawn) for at least 1 month and were fed small insects 4 times each week. Ants were collected freshly from natural colonies as required. We also included a non-predatory stinkbug, *Plautia affinis* (Fig. 1E), and these were obtained from orange orchards in Somersby, NSW. The non-predator was used in this study as a positive control against the possibility that the flies simply respond in a generic way to olfactory cues from any insect or spider source. Female spiders were used in our study because they were more abundant than male spiders, while worker ants were selected as they are the colony members that forage and attack prey.

### Olfactory cues from predators and non-predators

Olfactory cues from predators or non-predators were obtained by blowing charcoal filtered air over predators or non-predators into arenas. Briefly, a 50 mL closed glass volatile collection chamber (Sigma-Aldrich, USA), with an inlet and outlet, containing a single spider, a group of 6 ants, a non-predator, or no insect (filtered air control) was set-up 30 min before each experiment to allow a buildup of olfactory cues within the chamber. After 30 min, charcoal filtered air was passed through the chamber to carry olfactory cues from the volatile collection chamber into the test arenas using a gas sampling pump (KNF Pumps, Model no. NMP850.1.2KNDCB, Switzerland) at a rate of 1 L/min.

### Arenas and software

We designed two kind of arenas for this study. A behavioural arena (Fig. S1A) was used for analysis of motility, foraging and mating, while an oviposition arena was used to assess oviposition behaviour. The behavioural arena comprised of a closed, clear polystyrene Petri dish (145 mm dia. x 20 mm deep). The Petri dish was covered on all sides with white lamination paper (100 mm high) to mitigate possible positional biases caused by external visual stimuli. The arena had 2 holes (5 mm dia.) on the sides for inlet and outlet of olfactory cues from insects or filtered air. Video recordings were carried out with an overhead HD camera (Go Video, Digital 540TLV) at recording speed of 25 frames per second. The arena was placed 1 m below the camera and was lit by fluorescent lights, although recordings of mating, which occurs at dusk^48^, were enabled using infrared lighting. The camera was connected to a digital video recorder and each recording was for 10 min.

The oviposition arena (Fig. S1B) was a cylindrical clear polystyrene jar (150 mm high x 90 mm dia.). Two holes (5 mm dia.) on the sides of the jar served as inlet and outlet for olfactory cues or filtered air. An Eppendorf tube (5 mL) with 10–12 holes (<1 mm dia.) on the upper half served as oviposition device. To stimulate egg laying, the oviposition device contained 1 mL of mango juice, which contains the known *B. tryoni* oviposition stimulant γ‐octalactone^49^. After each trial, the arena was washed with warm water, wiped with 70% ethanol and air dried for 20 min. Motility (active time, mean velocity, distance moved), time spent in zones, number of visits to zones and x, y coordinates of flies was quantified using Lolitrack Ver 4 (Loligo Systems, Denmark). Mating behaviour was recorded using BORIS V6.3.4^50^. Thirty replicates were conducted for each bioassay for each predator.

### Bioassays

For motility assays, a 10-day old virgin male or female fly was placed in the arena and was allowed to acclimatize for 20 min, after which one of the olfactory cues or filtered air was pumped into the arena through the inlet. Fly movement was recorded for 10 min.

The foraging assay was conducted following Zaninovich *et al.*^51^ with minor changes. A single virgin 10-day old male or female fly that had been provided water, but no food for 24 hours was introduced into the arena and allowed to acclimatize for 20 min. Next, filtered air or air containing one of the olfactory cues from predators or non-predator was pumped through the inlet of the arena for 1 min before dispensing 100 µL of sugar solution (commercial cane sugar, 10% w/v) using a micropipette onto the centre of the arena demarcated as ‘food zone’. The food zone consisted of a Petri dish (50 mm dia.) containing the sugar solution. The number of visits made, and time spent by flies in the food zone was recorded for 10 min and analyzed.

Mating assays were carried out at dusk, the normal mating time of *B. tryoni*. A pair of 15-day old virgin male and female flies were introduced into a behavioural arena 30 min before the onset of dusk. They were allowed to acclimatize for 20 min after which one of the olfactory cues or filtered air was pumped through the inlet of the arena. The activity of flies was recorded over-night and the video was analyzed using BORIS software. Number of copulation attempts and copula duration was recorded.

Oviposition assays were conducted using the oviposition arena. A single 15-day-old gravid female was introduced into the arena and was allowed to acclimatize for 20 min. An oviposition device was placed on the floor in the centre of the arena. Simultaneously, one of the olfactory cues or filtered air was pumped through the inlet of the arena. The flies were allowed to oviposit for ~16 h. The collected eggs were washed into a Petri dish and counted under a stereo microscope (MZ6, Leica Microsystems, Germany).

### Statistics

Data for motility, foraging and oviposition assays did not conform to a normal distribution, and so were subjected to Kruskal-Wallis test followed by Dunn’s multiple comparision test. Statistics were calculated using GraphPad Prism version 7 for Mac (GraphPad Software, San Diego, CA, USA). Data for mating behaviour, including number of copulation attempts and copula duration, were subjected to Welch ANOVA. Data comparing number of flies that did and did not attempt copulation or mate in different treatment groups were subjected to Fishers Exact Tests.

## Data Availability

The datasets generated and analysed during this current study are available from the corresponding author on reasonable request.

## Supplemetary information

Supplemetary information.
