# Supplementary material for: Forewarned is forearmed: Queensland fruit flies detect olfactory cues from predators and respond with predator-specific behaviour
Source: Sci Rep. 2020 Apr 29;10:7297. doi: 10.1038/s41598-020-64138-6 (PMC7190731; doi:10.1038/s41598-020-64138-6)
Supplement: Supplementary file 1 — Supplemetary information. [file 41598_2020_64138_MOESM1_ESM.pdf]

Forewarned is forearmed: Queensland fruit flies detect olfactory cues from predators and responds with predator-specific behaviour

Vivek Kempraj*, Soo Jean Park and Phillip W. Taylor

Applied BioSciences, Macquarie University, Sydney, NSW, Australia
*Corresponding author: vivek.kemparaju@hdr.mq.edu.au

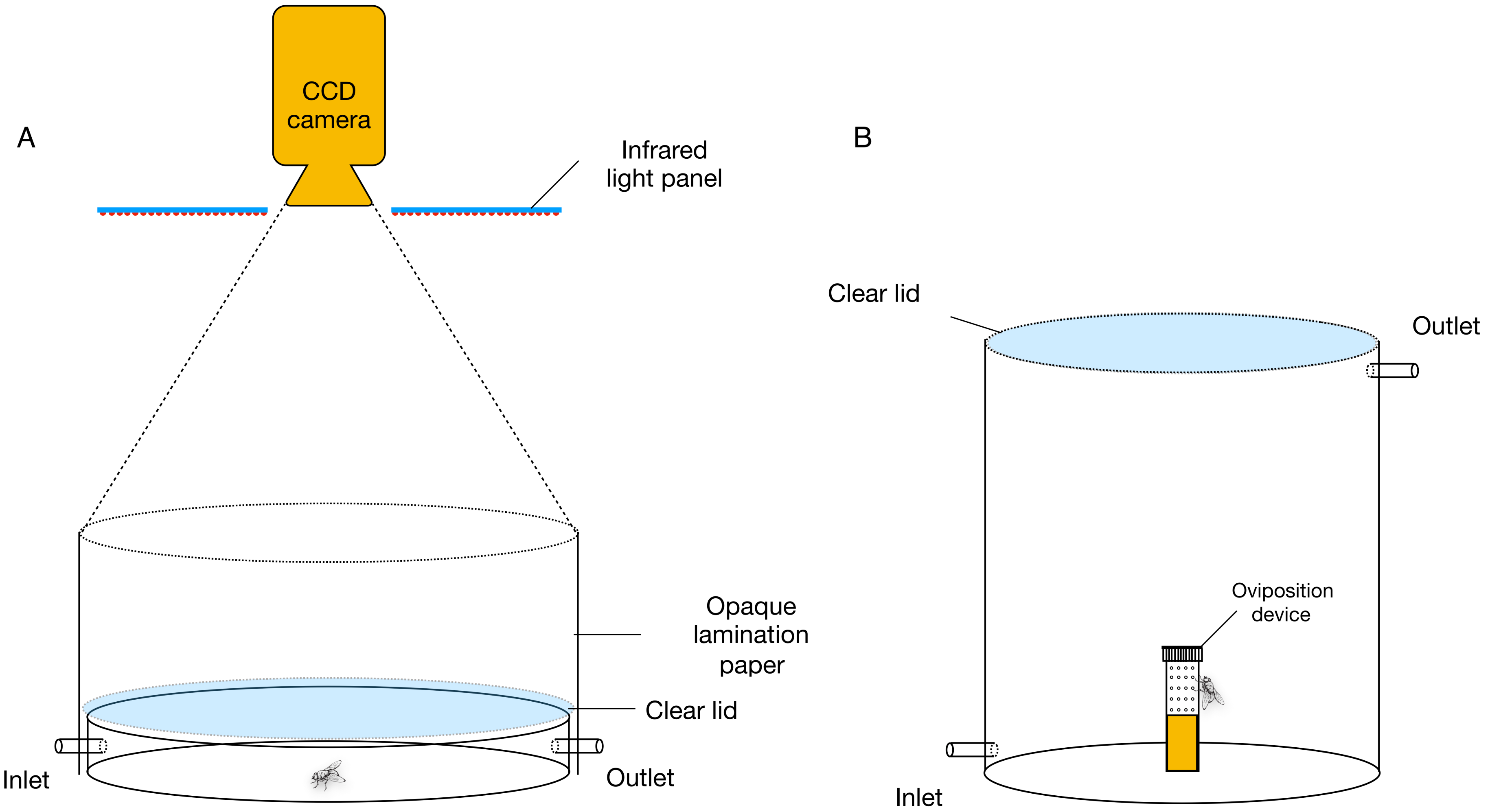
A
CCD camera
Infrared light panel
Opaque lamination paper
Clear lid
Inlet
Outlet
B
Clear lid
Outlet
Oviposition device
Inlet
